# Interplays of mutations in *waaA*, *cmk*, and *ail* contribute to phage resistance in *Yersinia pestis*

**DOI:** 10.3389/fcimb.2023.1174510

**Published:** 2023-05-26

**Authors:** Lisheng Xiao, Zhizhen Qi, Kai Song, Ruichen Lv, Rong Chen, Haihong Zhao, Hailian Wu, Cunxiang Li, Youquan Xin, Yong Jin, Xiang Li, Xiaoqing Xu, Yafang Tan, Zongmin Du, Yujun Cui, Xuefei Zhang, Ruifu Yang, Xilin Zhao, Yajun Song

**Affiliations:** ^1^ Laboratory of Molecular Vaccinology and Molecular Diagnostics, School of Public Health, Xiamen University, Xiamen, China; ^2^ School of Basic Medicine, Anhui Medical University, Hefei, China; ^3^ State Key Laboratory of Pathogen and Biosecurity, Beijing Institute of Microbiology and Epidemiology, Academy of Military Medical Sciences (AMMS), Beijing, China; ^4^ Qinghai Institute for Endemic Disease Prevention and Control, Xining, China; ^5^ National Health Commission - Qinghai Co-construction Key Laboratory for Plague Control, Xining, China; ^6^ Hua Dong Research Institute for Medicine and Biotechniques, Nanjing, China; ^7^ Department of Laboratory Medicine, First Medical Center of Chinese People’s Liberation Army (PLA) General Hospital, Beijing, China

**Keywords:** *Yersinia pestis*, phage, phage resistance, fitness cost, *waaA*, *cmk*, *ail*

## Abstract

Plague caused by *Yersinia pestis* remains a public health threat worldwide. Because multidrug-resistant *Y. pestis* strains have been found in both humans and animals, phage therapy has attracted increasing attention as an alternative strategy against plague. However, phage resistance is a potential drawback of phage therapies, and the mechanism of phage resistance in *Y. pestis* is yet to be investigated. In this study, we obtained a bacteriophage-resistant strain of *Y. pestis* (S56) by continuously challenging *Y. pestis* 614F with the bacteriophage Yep-phi. Genome analysis identified three mutations in strain S56: waaA* (9-bp in-frame deletion _249_GTCATCGTG_257_), cmk* (10-bp frameshift deletion _15_CCGGTGATAA_24_), and ail* (1-bp frameshift deletion A_538_). WaaA (3-deoxy-D-manno-octulosonic acid transferase) is a key enzyme in lipopolysaccharide biosynthesis. The waaA* mutation leads to decreased phage adsorption because of the failure to synthesize the lipopolysaccharide core. The mutation in *cmk* (encoding cytidine monophosphate kinase) increased phage resistance, independent of phage adsorption, and caused *in vitro* growth defects in *Y. pestis.* The mutation in *ail* inhibited phage adsorption while restoring the growth of the *waaA* null mutant and accelerating the growth of the *cmk* null mutant. Our results confirmed that mutations in the WaaA–Cmk–Ail cascade in *Y. pestis* contribute to resistance against bacteriophage. Our findings help in understanding the interactions between *Y. pestis* and its phages.

## Introduction

Phages have long been used in medicine to identify specific bacteria or as alternative treatment for treating bacterial diseases. Because of the global emergence of antibiotic resistance, phage treatment has attracted great attention as an alternative or complement to antibiotic therapy. *Y. pestis*, the causative agent of plague, is transmitted by fleabite or respiratory droplets (Gage and Kosoy, 2005; Cathelyn et al., 2006). Three plague pandemics have been recorded in history, resulting in approximately 200 million deaths (Sun and Singh, 2019). Although human plague cases have been well controlled in most countries, sporadic cases or outbreaks are occasionally reported in plague foci worldwide. In 2017, a pneumonic plague outbreak in Madagascar caused 209 deaths in 4 months (Tsuzuki et al., 2017). Antibiotics are generally effective in the treatment of plague, but several antibiotic-resistant strains of *Y. pestis* have been isolated from patients and rodents in recent years (Cabanel et al., 2018; Sebbane and Lemaitre, 2021). In this context, bacteriophage therapy has been suggested as an alternative, the efficacy of which depends on the administration route (e.g., oral, intramuscular, and aerosol spray) or the frequency of phage application (Anisimov and Amoako, 2006; Sebbane and Lemaitre, 2021; Vagima et al., 2022).

The use of phage therapy has many ethical considerations. For instance, discussing the known and unknown risks of phage therapy can be challenging for patients and physicians. Moreover, the development of phage therapy has been hindered by the lack of predefined regulatory pathways for phage production and concerns about intellectual property protection (Kingwell, 2015). However, the main limitation of phage therapy is phage resistance. In natural environments, bacteria are subjected to strong selective pressure by bacteriophages (Vlot et al., 2018). In response, bacteria have developed mechanisms to resist phages, including inhibition of DNA injection, restriction and modification, abortive infection, CRISPR–Cas, and inhibition of adsorption via phase variation of cell surface receptors (Kim et al., 2015; Takeuchi et al., 2016; Reyes-Robles et al., 2018). Although the CRISPR–Cas system has been studied for decades, unknown defense systems in bacteria remain to be discovered (Makarova et al., 2013). For example, Doron et al. have identified several unreported defense systems using microbial pangenome analysis (Doron et al., 2018). Notably, mutations in certain genes may render the host bacterium resistant to phage infection (Laanto et al., 2020).

In this study, we used *in vitro* culture assays to screen *Y. pestis* strains with varying degrees of phage resistance and obtained several strains with mild-to-complete resistance to bacteriophages. We examined heritable gene mutations in resistant strains and identified *Y. pestis* genes involved in resistance to phage lysis, such as *waaA*, *cmk*, and *ail*. *waaA* encodes 3-deoxy-D-manno-octulosonic acid transferase, which is involved in the synthesis of lipopolysaccharide (LPS) and serves as the phage receptor in *Y. pestis* (Kiljunen et al., 2011). *cmk* encodes cytidine monophosphate (CMP) kinase, which catalyzes the transfer of a phosphoryl group from ATP to CMP or dCMP and plays a crucial role in the biosynthesis of nucleoside precursors (Walker et al., 2012). *Escherichia coli* with the *cmk* mutation are less susceptible to T7 phage (Qimron et al., 2006). Our previous study indicated that Ail contributes to phage adsorption in *Y. pestis* by interacting with the phage tail fiber protein (Zhao et al., 2013). By deciphering the evolutionary processes of *Y. pestis* against phage lysis stress, we have provided insights into the physiological processes involved in receptor recognition, phage DNA replication, and host degradation resistance.

## Materials and methods

### Bacterial strains, plasmids, phage, and media

The *Y*. *pestis* strains used in this study are described in **Table 1**. Other bacterial strains and plasmids are described in **Supplementary Table S1**, and the primers used in the study are described in **Supplementary Table S2**. The phage Yep-phi used in this study can effectively lyse all tested Chinese isolates of *Y. pestis* but is unable to lyse other *Yersinia* species (Zhao et al., 2011). *Y. pestis* and Yep-phi cultures were incubated at 26°C and *E. coli* at 37 °C. Luria–Bertani (LB) media were used for bacterial liquid cultures. A soft agar medium was prepared by adding 0.4% (wt/vol) agar to liquid media. Plates were supplemented with ampicillin (Amp, 100 μg/ml), kanamycin (Kan, 100 μg/ml), spectinomycin (Spe, 100 μg/ml), or chloramphenicol (Cm, 34 μg/ml) when required.

**Table 1:** *Y*. *pestis* strains used in this work.

Strains	Characteristics
614F	Antiqua biovar strain
S12	Partially phage-resistant strain after 12 passages of 614F with phage challenging, with _249_GTCATCGTG_257_ deletion in *waaA* gene, accession numbers SAMN07501781
S38	Phage-resistant strain after 38 passages of 614F with phage challenging, with _15_CCGGTGATAA_24_ deletion in *cmk*
S56	Phage-resistant strain after 56 passages of 614F with phage challenging, with A_538_ deletion in the *ail* gene on top of S38 accession numbers SAMN07488721
S88	Phage-sensitive strain after 56 passages of 614F without phage challenging, accession numbers SAMN07488727
201	Microtus biovar strain
Δ*waaA*	*waaA* was replaced by a Spe cassette in 201
Δ*cmk*	*cmk* was replaced by a Spe cassette in 201
Δ*ail*	*ail* was replaced by a Spe cassette in 201
waaA*	*waaA* (Δ_249_GTCATCGTG_257_), Δ*waaA* with Spe cassette replaced by the *waaA* of S56
cmk*	*cmk* (Δ_15_CCGGTGATAA_24_), Δ*cmk* with Spe cassette replaced by the *cmk* of S56
ail*	ail* (ΔA_538_), Δ*ail* with Spe cassette replaced by the *ail* of S56
waaA*/cmk*	cmk*/Δ*waaA* with Spe cassette replaced by the *waaA* of S56
waaA*/ail*	ail*/Δ*waaA* with Spe cassette replaced by the *waaA* of S56
cmk*/ail*	ail*/Δ*cmk* with Spe cassette replaced by the *cmk* of S56
waaA*/cmk*/ail*	cmk*/ail* with the *waaA* in 201 replaced by the *waaA* of S56
C_waaA*	Complemented with *waaA* of 201 on pACYA184
C_cmk*	cmk* with overexpressed *cmk* on pBAD33

### Identification of phage-resistant *Y. pestis* 614F derivates

Different titers (from 1.5 × 10^8^ plaque-forming unit [PFU] to 1.5 × 10^4^ PFU) of 0.5 ml strain 614F (10^9^ CFU/ml) and 5 ml Yep-phi were mixed and incubated at 26°C for 24h. The serial mixtures were separately plated on LB agar and incubated at 26°C for 24h. Colonies on plates were resistant to the corresponding titer of phage and were selected for passages. Bacterial stocks of every passage were stored for future analysis. Coculture passages were performed to obtain a fully resistant Yep-phi derivate of 614F (phage titer 1.5 × 10^8^ PFU). The genomes of resistant and wild-type 614F strains were sequenced and compared. The identified mutations were screened with mismatch–polymerase chain reaction (PCR) in all passage stocks to determine when these mutations occurred during the entire passage process (**Supplementary Table S2**) (Wangkumhang et al., 2007).

### Mutant construction

Scarless genome editing was used to generate gene knockouts and point mutants in *Y. pestis* (Kim et al., 2014). In brief, selection cassettes containing Spe-resistant genes, flanked by FRT, I-SceI sites, and 120-bp homologous arms, were electroporated into 201/pREDTKI, which encodes meganuclease I-SceI under the control of the an hydrotetracycline-induced *tetA* promoter and λ-Red recombinase genes under the control of the arabinose-inducible *araB* promoter as well as a temperature-sensitive replication origin. The knockout strains were confirmed via PCR and sequencing. Using the primers listed in **Supplementary Table S2**, the PCR fragment containing the SacI/HindIII site of mutant gene S56 was amplified and cloned into the donor plasmid pKSI-1, which with I-SceI recognition sites was digested with SacI and HindIII to produce pKSI-1_waaA*, pKSI-1_cmk*, and pKSI-1___ ail*. The donor plasmid was electroporated into knockout strains; transformants were restruck onto LB plates containing Kan, Spe, and Amp and grown overnight at 26°C. Restruck colonies were suspended in LB containing Kan, Spe, and Amp and grown overnight at 26°C. The colonies were repatched twice onto LB supplemented with 10 mmol L-arabinose for inducing lambda red recombinase and 20 mmol IPTG for inducing I-SceI expression to loop out the selection cassette and donor plasmid at 26°C. To verify the loss of the selection cassette, isolates were tested for growth on LB plates with or without Spe and Amp. The sensitive isolates were screened via PCR and sequencing to confirm mutagenesis. Successive passage was performed at 37°C to remove pREDTKI from the strain. The waaA*/cmk*/ail* mutant was constructed via suicide plasmid-mediated genome editing (Philippe et al., 2004). Homologous upstream and downstream fragments flanking the waaA* mutation were amplified from S56 using PCR. The fragments were cloned into the suicide plasmid pDS132, which was digested with SacI and SalI, and replicated in S17-1 λpir. The recombinant plasmids were purified and introduced in cmk*/ail* via conjugation. Plates containing Cm were screened after incubation at 37°C overnight. The clone was selected on a plate containing 7% sucrose following growth at 26°C for 4 days. We selected transformants that grew on plates but not on Cm plates. The strain containing waaA*/cmk*/ail* was detected via PCR and confirmed via sequencing.

### Growth curves

The overnight grown strains were cultured to OD_620_ of approximately 1.0, diluted 1:100 into 20 ml of LB, and cultured at 26°C and 200 rpm. OD_620_ was determined every 2h using 300 μl of bacterial culture. The OD_620_ values were used to draw growth curves. Data were obtained in triplicate, and experiments were repeated twice. Area under the curve analysis was used to quantify the differences in growth (Vornhagen et al., 2019).

### Phage infection assay

The lysis activity of each phage strain was examined using a spot test on *Y. pestis* (Paul et al., 2011; Zhao et al., 2013). In brief, 100 ml of bacteria cultured to exponential phase (OD_620_~1.0) were concentrated 10-fold via centrifugation and mixed with 3 ml of liquefied soft agar medium. The mixture was plated on LB plates to create double-layer agar plates. When the medium solidified, 3 μl of serial dilutions of Yep-phi were spotted on the plates. Plates were incubated at room temperature for 10 h and then examined and photographed.

### Phage adsorption assay

For phage adsorption, approximately 8 × 10^5^ PFU of Yep-phi in 100 μl was mixed with 500 μl bacteria (OD_620_~1.0). LB was used as a nonadsorbing control in each assay, and the phage titer in the control supernatant was set to 100%. The mixture was incubated at room temperature for 5 min and centrifuged at 16,000 × *g* for 3 min. Residual PFU percentage was calculated as described in a previous study (Kiljunen et al., 2011).


$$\text{Residual PFU }=\;\frac{\text{number of PFU in mixture}}{\text{number of PFU in LB}}\;\times \;100\%$$

Statistical differences were determined using one-way analysis of variance (ANOVA) with three independent data sets. To reveal the temporal phage adsorption kinetics of all strains, residual PFU percentage was tested at different time points (2, 5, 10, and 15 min). Each assay was performed in triplicate and repeated twice. Statistical differences were determined using two-way RM ANOVA with Dunnett’s multiple comparisons to wild-type groups.

### Phage efficiency of plating assay

To determine the efficiency of plating (EOP) of Yep-phi on different strains, 300 μl of wild-type and mutant *Y. pestis*–cultured bacteria (OD_620_~1.0) were mixed with 100 μl of Yep-phi in 3 ml of 0.4% soft agar and poured onto LB plates. The number of PFUs was counted after 24h–48h. Each strain was verified in triplicate. EOP was calculated as described (Hyman and Abedon, 2010).


$$\text{EOP }=\;\frac{\text{number of PFU on mutant strain}}{\text{number of PFU on wild}-\text{type}}\;\times \;100\%$$

### Isolation and analysis of LPS

LPS isolation was performed using the phenol–water extraction method (Zhang and Skurnik, 1994). In brief, 9 ml overnight bacterial cultures were collected via centrifugation and resuspended in 1.5 ml distilled water. The bacterial suspensions were incubated at 70°C for 1h and then mixed with water-saturated phenol (pH 4.0) reheated to 70°C for 10 min and then transferred to ice for cooling (< 10°C). Then, the cells were centrifuged at 2,000 × *g* for 20 min. The uppermost aqueous layer obtained was transferred to a new Eppendorf tube and 2 volumes of acetone were added to precipitate LPS. The LPS pellet was dissolved in 150 μl of water, of which 5 μl of LPS was analyzed via 15% sodium dodecyl sulfate-polyacrylamide gel electrophoresis and silver staining (Zhang and Skurnik, 1994).

### Protein expression and purification

The PCR-generated DNA fragment comprising *waaA* and waaA* was cloned into the BamHI/HindIII site of the pET32a vector. The plasmid was introduced into DH5α cells, and its DNA sequence was confirmed. The plasmid was extracted and introduced into *E. coli* BL21(DE3) cells. To induce the expression of WaaA and WaaA* in *E. coli* BL21(DE3) cells, 1 mM IPTG was added when the culture reached the mid-exponential growth phase at an OD_600_ of approximately 0.8. The bacterial cells were cultured at 37°C for 4h and collected. WaaA and WaaA* were purified via immobilized metal affinity chromatography on a nickel cephalosporin HP column. The proteins were desalted with PBS (pH 7.2) and 20% glycerol and concentrated using a protein concentration column (Mamat et al., 2009). Protein concentrations were determined using a BCA protein assay kit.

### Circular dichroism spectroscopy

To compare the circular dichroism (CD) spectra of wild-type WaaA and WaaA*, a Jasco J-815 spectropolarimeter (Greenfield, 1999; Kelly et al., 2005) was used. Protein solutions (0.1–0.35 mg/ml) were quantified at 200–260 nm. Spectra were obtained with a scan speed of 50 nm/min and 0.5-nm data pitch. The percentage of secondary structure was estimated using online software connected to Jasco J-815.

### Protein structure analysis

Protein Data Bank (PDB) was used to conduct homology search (Rose et al., 2015). For WaaA, the structure of membrane-embedded monofunctional glycosyltransferase WaaA from *Aquifex aeolicus* (PDB code: 2XCI.A) was used as the template based on the quality of models produced. Multiple sequence alignment was performed using Clustal Omega from UniProt (Yip et al., 2008). The ESPript server was used to predict the secondary structure of WaaA (Robert and Gouet, 2014). The Alphafold v2.3.1 monomer_casp14 model was used to predict the structures of WaaA and WaaA* (Jumper et al., 2021). The biological and functional insights derived from the predicted models were verified by matching the models with the protein function database.

## Results

### Continuous phage challenge produced phage-resistant derivates of strain 614F

**Figure 1:**
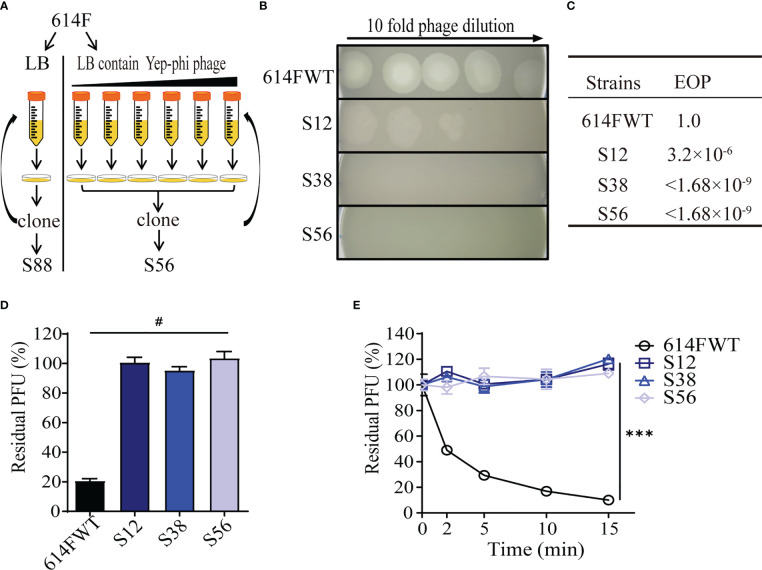
Screening and characterization of phage-resistant mutants of strain 614F. **(A)** Screening procedures. **(B)** Ten-fold dilution of lysates of Yep-phi applied to bacterial lawns of 614F wild-type and phage-resistant strains. The maximum titer of bacteriophage is 10^8^ PFU. **(C)** The efficiency of plating (EOP) of Yep-phi phage on various strains. EOP = (number of PFUs on mutant strain)/(number of PFUs on wild-type strain). The titer of bacteriophage is 1.68 × 10^9^ PFU. **(D)** Adsorption of Yep-phi on strain 614F and its derivates. The *Y*-axis represents percentage of residual plaque-forming unit (PFU). Error bars show standard deviations of the mean of three biological replicates. Significance was determined by one-way ANOVA followed by Dunnett’s multiple comparison test. ^#^ *P<* 0.0001. **(E)** Adsorption kinetics of Yep-phi to strain 614F and its derivates. ***P< 0.001 (two-way RM ANOVA with Dunnett’s multiple comparison test). Error bars indicate standard deviations in triplicate samples.

Yep-phi is routinely used as a diagnostic phage for identifying *Y. pestis*, and no natural Yep-phi phage-resistant *Y. pestis* isolate has been reported (Zhao et al., 2011). In this study, we exposed cultures of *Y. pestis* 614F to serially diluted phage Yep-phi. The surviving clones were selected and amplified continuously. **Figure 1A** illustrates how resistant mutants were identified by rounds of progressive challenges with different phage titers. Phage spotting assays revealed that after 12 rounds of phage challenge, the derivate of 614F (strain S12) showed decreased phage susceptibility (resistant to phage titer 10^5^ PFU), whereas later derivates (S38 and S56, with 38 and 56 rounds of challenges, respectively) were fully resistant to phage titer 10^8^ PFU **(** **Figure 1B** **)**. The EOP assay showed a similar result; the EOP value for S12 was 3.2 × 10^−6^, whereas the EOP values for S38 and S56 were<1.68 × 10^−9^ (**Figure 1C**). Our results confirmed that the 614F strain gradually developed full resistance to phage Yep-phi under continuous stress. Residual PFU percentages were used to evaluate the adsorption capability of 614F and its derivates S12, S38, and S56. All derivate strains lost their ability to adsorb phages (**Figure 1D**), which was confirmed through the temporal kinetics of adsorption assays (**Figure 1E**).

### Genomic analysis revealed mutations in phage-resistant strain S56

To examine the genetic variations responsible for phage resistance, we sequenced the genomes of strain S56 and its ancestor strain 614F. A control strain S88 (56 passages without phage challenges) was sequenced to rule out unrelated mutations. We identified three mutations in S56 (**Supplementary Figure S1**): a 9-bp in-frame deletion (_249_GTCATCGTG_257_) in *waaA* (gene ID: YPO0055), a 10-bp frame shift deletion (_15_CCGGTGATAA_24_) in *cmk* (gene ID: YPO1391), and a 1-bp frame shift deletion (A_538_) in *ail* (gene ID: YPO2905), which are annotated according to *Y. pestis* CO92. The mutations were named waaA*, cmk*, and ail*, respectively, and mismatch-PCR was used to screen their presence in passage stocks. The findings revealed that waaA* occurred in passage 12 (strain S12), cmk* occurred in passage 38 (strain S38) in addition to waaA*, and ail* occurred in passage 56 (strain S56) in addition to waaA*/cmk*.

### waaA*/cmk*/ail* mutations reproduced phage resistance phenotypes in strain 201

**Figure 2:**
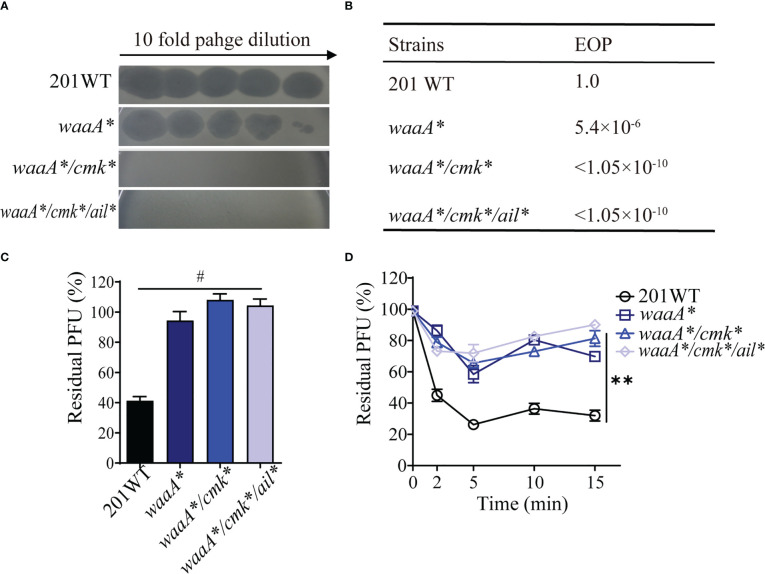
Reproduction of phage resistance induced by *waaA*, *cmk*, and *ail* mutations in strain 201. **(A)** Ten-fold dilution of Yep-phi lysates applied to lawns of 201 wild-type and mutant bacterial strains. The maximum titer of bacteriophage is 3.15 × 10^7^ PFU. **(B)** Efficiency of plating (EOP) for different strains. The titer of bacteriophage is 1.05 × 10^10^ PFU. **(C)** Adsorption of 8 × 10^5^ PFU Yep-phi to *Y. pestis* 201 WT and its single-, double-, or triple-gene mutants shown as residual PFU percentages. Significance was determined by one-way ANOVA. ^#^ *P<* 0.0001. **(D)** Adsorption kinetics of Yep-phi to wild-type *Y. pestis* 201 and its mutants (ca. 2 × 10^6^ PFU/10^8^ CFU). **P< 0.01 (Dunnett’s multiple comparison test of two-way RM ANOVA).

To determine whether a similar phage-resistant profile can be reproduced and the roles of mutations in *waaA* (9-bp deletions), *cmk* (10-bp deletions), and *ail* (1-bp deletion), we constructed single, double, or triple mutants derived from strain 201. Strain waaA* was partially resistant to phage Yep-phi (resistant to 3.15 × 10^3^ PFU, **Figure 2A**), with an EOP value of 5.4 × 10^−6^ (**Figure 2B**, phage titer 1.05 × 10^10^ PFU). Moreover, waaA*/cmk* and waaA*/cmk*/ail* were fully resistant to phages with EOP< 1.05 × 10^−10^, which is much lower than that for waaA* (**Figure 2B**). Adsorption assays indicated that all mutants had a significantly lower ability to adsorb phage Yep-phi, and the residual PFU percentages among the three mutants were indistinguishable (**Figures 2C, D**). These results suggest that the waaA* mutation affects the binding between *Y. pestis* and its phage.

### The waaA* mutation inhibits phage adsorption by truncating LPS

**Figure 3:**
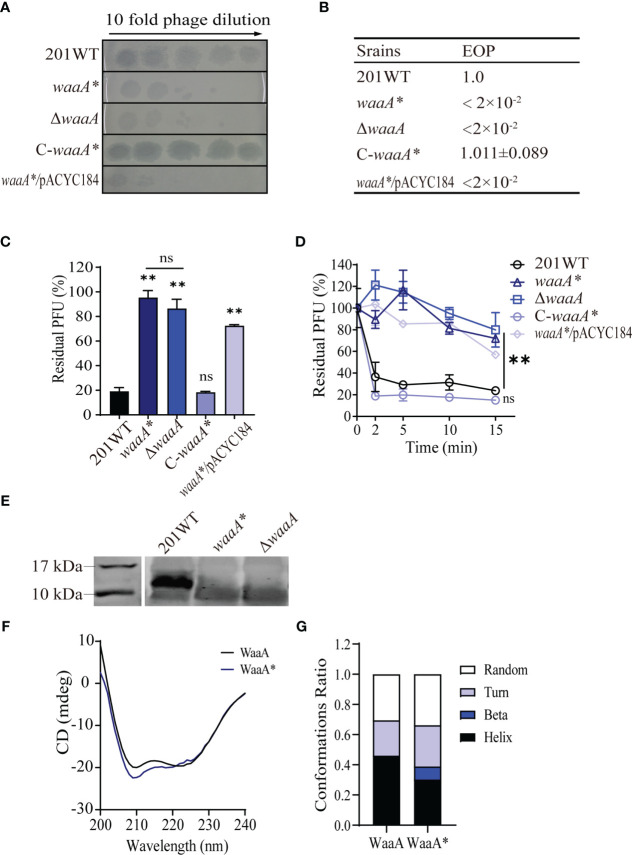
The *waaA* mutation inhibits phage adsorption by disrupting LPS. **(A)** Ten-fold dilution of Yep-phi lysates applied to bacterial lawns of wild-type and mutant *Y. pestis* strains. The maximum titer of bacteriophage is 4.35 × 10^7^ PFU. **(B)** Efficiency of plating (EOP) of 2 × 10^2^ PFU phage for different strains. **(C)** Adsorption of Yep-phi to *Y. pestis* 201 and *waaA* mutant strains, expressed as a percentage of residual PFU. Significance was determined by one-way ANOVA; **P< 0.01. ns, non-significant. **(D)** Adsorption kinetics of Yep-phi to *Y. pestis* and *waaA* mutant strains expressed as percentages of residual PFU. Two-way RM ANOVA was used to determine significance between the mutant and wild-type groups; ** *P<* 0.01. **(E)** Analysis of isolated LPS fractions from *Y. pestis* 201 and waaA mutants using SDS/PAGE. The gel was silver stained for visualization. **(F)** CD spectra of WaaA and WaaA*. **(G)** The build-in software of the spectropolarimeter predicted the conformations ratio between WaaA and WaaA* of *Y. pestis* 201.

Phage adsorption defects were found in strain waaA* and strain 614F carrying the waaA* mutation. The waaA* mutant and the *waaA* null mutant Δ*waaA* displayed similarly decreased phage susceptibility (resistant to 4.35 × 10^3^ PFU phage), with decreased EOP (**Figure 3A**). In the complementation test, the phage susceptibility of waaA* supplemented with plasmid pACYC184_*waaA* was restored and comparable to that of wild-type strain 201 (**Figures 3A, B**). In the adsorption kinetics assays, the phage adsorption ability of waaA* was similar to that of Δ*waaA*, and waaA* mutant complemented with plasmid pACYC184_*waaA* showed similar phage adsorption ability to the wild-type strain, confirming the anti-adsorption effect of the waaA* mutation (**Figures 3C, D**).

Because the *waaA* gene, encoding 3-deoxy-D-mannooctulosonic acid (KDO) transferase, is involved in LPS synthesis (Chung and Raetz, 2010), we hypothesize that the mutation in waaA* affects the adsorption of Yep-phi phage because of failure to synthesize LPS, which is the phage adsorption receptor. At 26°C, *Y. pestis* expresses rough LPS, which does not contain the *O*-polysaccharide that is considered the phage receptor (Kiljunen et al., 2011). Rough LPS was expressed in 201 WT but was not stained on the gel by silver (a characteristic of a core-lacking LPS) in either Δ*waaA* or waaA* (**Figure 3E**), which confirmed the failure of complete LPS core synthesis in the two mutants (Meredith et al., 2006; Dentovskaya et al., 2011). CD spectra assays demonstrated that wild-type WaaA has two negative absorption bands at 209 and 222 nm, which are characteristic of α-helical proteins, and the CD spectrum of WaaA* protein is slightly different from that of wild-type strain (**Figure 3F**). Meanwhile, the build-in software of the spectropolarimeter predicted decreased α-helix and increased β-sheet in WaaA* protein comparing with the wild-type WaaA (**Figure 3G**).

The 9-bp (_249_GTCATCGTG_257_) in-frame deletion in *waaA* resulted in the _84_TMT_86_ deletion in WaaA. To further determine the consequence of the _84_TMT_86_ deletion in WaaA, we compared the predicted structures of wild-type WaaA and mutated WaaA*. Sequence alignment of WaaA and WaaA* showed the presence of charged residue matching the monofunctional glycosyltransferase WaaA of *A. aeolicus* (PDB code: 2XCI.A) (Schmidt et al., 2012; Rose et al., 2015). In *A. aeolicus*, residues S54 and R56 of WaaA had been proven vital for its KDO transferase activity, and these two residues together with S28 and E31 provide necessary hydrogen bonds to bind tetraacyl-4'-phosphate lipid A during KOD transferring process as part of the receptor-substance binding site of WaaA (Schmidt et al., 2012). Secondary structure predictions of WaaA and WaaA* indicated that the _84_TMT_86_ deletion occurred in the junction of β-sheet 2 (β2) and α-helix3 (α3), right next to residues S54 and R56 of *A*. *aeolicus* WaaA (**Supplementary Figure S2**). Sequence alignment of multiple bacterial WaaA showed that the _84_TMT_86_ region is conserved among multiple bacterial species (**Supplementary Figure S3**). Additionally, Alphafold analysis of WaaA and WaaA* proteins demonstrated that their predicted structures are slightly different from each other (**Supplementary Figures S4A–C**). The _84_TMT_86_ is located at an α helix-loop-β turn connection in WaaA protein, and the deletion of _84_TMT_86_ of resulted in a shorter length of the α helix, followed by a shift of approximately 2.7 Å in the loop and a 6.8° deflection of the in the β turn (**Supplementary Figure S4D**).

Our results suggest that the _84_TMT_86_ deletion in WaaA hinders its binding to the precursor of lipid A, and causes the loss of its glycosyltransferase function. The three amino acids in-frame deletion in waaA* resulted in a core-lacking LPS of *Y. pestis*, leading to phage resistance similar to Δ*waaA*.

### cmk*-related phage resistance is independent of adsorption

**Figure 4:**
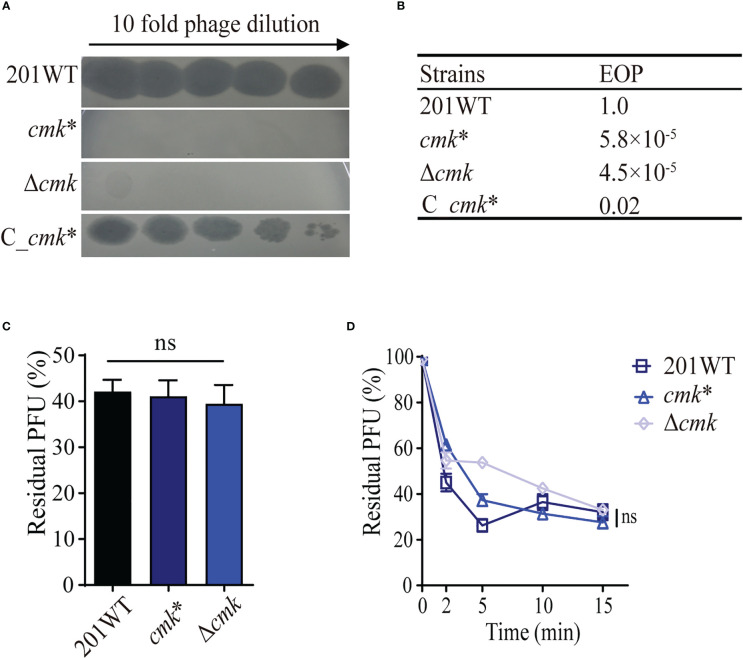
cmk* resists phage lysis without impacting phage adsorption. **(A)** Ten-fold dilution of Yep-phi lysates applied to bacterial lawns of wild-type and *cmk* mutant *Y. pestis* strains. The maximum titer of bacteriophage is 4.35 × 10^7^ PFU. **(B)** Efficiency of plating (EOP) for different strains. **(C)** Adsorption of Yep-phi to *Y. pestis* 201 and its *cmk* mutant strains expressed as a percentage of residual PFU. ns, non-significant (one-way ANOVA). **(D)** Adsorption kinetics of Yep-phi to *Y. pestis* and *cmk* mutant strains, expressed as percentages of residual PFU. ns, non-significant (Dunnett’s multiple comparison test of two-way RM ANOVA).

*cmk* encodes a cytidylate kinase that catalyzes phosphoryl transfer from ATP to (d)CMP (Tsao et al., 2015). Mutant waaA*/cmk* was more resistant to phage lysis than waaA*. However, the role of the *cmk* mutation against phage lysis remains unknown. To address this, we constructed cmk*, which contains a frameshift mutation (_15_CCGGTGATAA_24_ deletion). We performed infection assays using cmk*, C_cmk* (a complementary strain), and Δ*cmk* (*cmk* null mutant) simultaneously and found that cmk* and Δ*cmk* were resistant to a high phage titer (3.15 × 10^7^ PFU) (**Figure 4A**), with EOP values of 5.8 × 10^−5^ and 4.5 × 10^−5^, respectively (**Figure 4B**). C_cmk* restored susceptibility, comparable with wild-type 201. Notably, cmk* and Δ*cmk* have similar adsorption capacity compared with WT 201 (**Figures 4C, D**), suggesting that cmk*-mediated phage resistance is irrelevant for the adsorption of *Y. pestis* and phage particles. The 10-bp (_15_CCGGTGATAA_24_) deletion of *cmk* led to premature termination of Cmk translation. The mechanism of Cmk-related phage resistance needs further investigation.

### The ail* mutation affects the phage adsorption ability of *Y. pestis*

**Figure 5:**
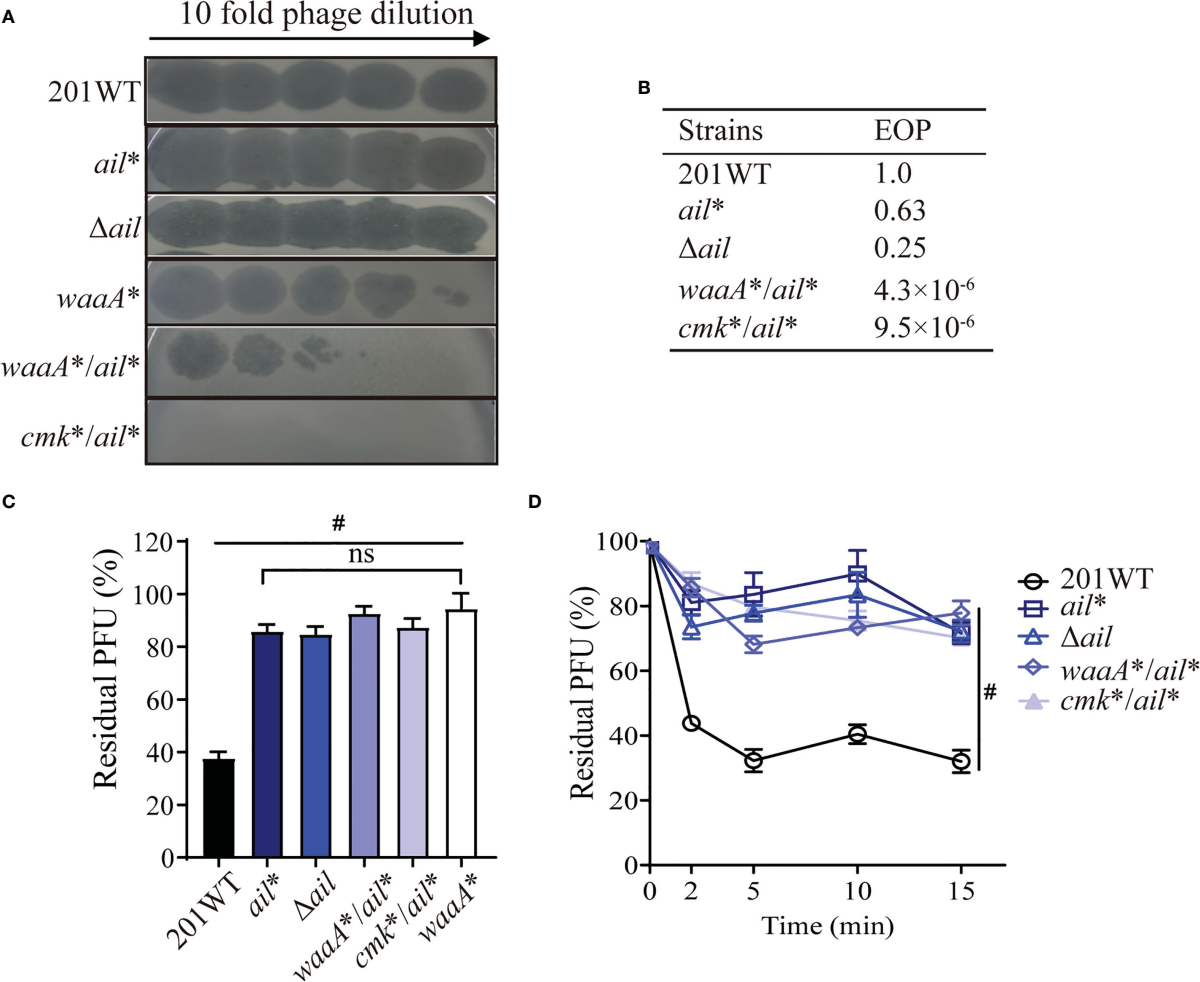
The *ail* mutation confers resistance to phages in *Y. pestis*. **(A)** Ten-fold dilution of Yep-phi lysates applied to bacterial lawns of wild-type and mutant strains. The maximum titer of bacteriophage is 4.35 × 10^7^ PFU. **(B)** Efficiency of plating (EOP) for different strains. **(C, D)** Yep-phi adsorption and adsorption kinetics to *Y. pestis* 201 and its mutant strains. ^#^ *P<* 0.0001. ns, non-significant.

In *Y. pestis*, Ail is an outer membrane protein that contains 182 amino acids. Our previous study revealed that Ail interacts with the phage tail fiber protein and contributes to phage adsorption in *Y. pestis* (Zhao et al., 2013). In this study, we identified the A_538_ deletion in *ail* in strain S56 (ail*, **Table 1**), which is at the very end of the *ail* coding region. ail* or Δ*ail* was as susceptible to phages as wild-type 201, with comparable EOP values (**Figures 5A, B**). Moreover, the ail* mutant showed similar phage adsorption defects as the null mutant Δ*ail* (**Figures 5C, D**). The addition of waaA* or cmk* to ail* did not make a difference in phage adsorption (**Figures 5C, D** **)**; however, it significantly increased resistance to phage compared with the ail* null strain **(** **Figures 4B**, **5B** **).** Our results indicate that ail* (1-bp deletion at A_538_) exhibited phage adsorption resistance, consistent with the effects of disrupting phage adsorption in Δ*ail*.

### Interplays of waaA*/cmk*/ail* mutations restore the growth of the mutants

**Figure 6:**
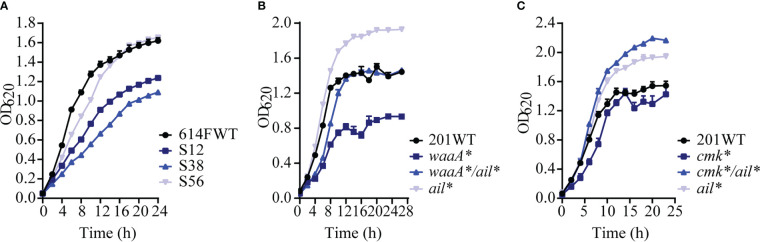
Growth attenuation caused by waaA* and/or cmk* mutations can be restored by ail* in *Y. pestis*. **(A)** Growth curve of *Y. pestis* 614F and its derivates. **(B)** ail* restored growth of waaA*. **(C)** ail* restored growth of cmk*. Data represent the average of three biological replicates; error bars indicate SEM.

The waaA*/cmk* mutant is resistant to high-titer phage attacks (**Figures 2A, B** **)**, and the ail* mutant is somewhat sensitive to phages even though it adsorbs phages less effectively. We wondered whether the evolution of phage resistance due to the waaA*/cmk* mutations was a liability that was compensated by the *ail* mutation or why the weakly lysis-resistant ail* mutation occurred in *Y. pestis* when the waaA*/cmk* mutation already renders the strain completely phage resistance. The 614F derivate S12 grows slower in LB than 614F. The growth of S38 is even slower than that of S12, which suggests the great fitness costs accompanying the phage resistance phenotype of these two derivates. Interestingly, S56 grew much better than S38, and its growth recovered to the level of 614F (**Figure 6A** and **Supplementary Figure S5A**). Because waaA*/cmk*/ail* mutations occurred sequentially in S12, S38, and S56, we assumed that waaA* and cmk* mutations influence the growth of the mutants in addition to conferring phage resistance onto them. Growth curves showed a deceleration of bacterial growth for cmk*, especially for waaA*, while the subsequent ail* mutation restored the mutant growth, as measured by calculating area under the curve (**Figures 6B, C** **;** **Supplementary Figures S5B, C**). Our results indicate that the growth deficiency in waaA*/cmk* mutants can be compensated by the A_538_ deletion of *ail.*

## Discussion

In this study, we obtained phage-resistant *Y. pestis* mutants by continuously challenging strain 614F with the Yep-phi phage. Characterization of these mutants identified successive mutations in *waaA*, *cmk*, and *ail*.

The *waaA* null mutant not only demonstrated resistance to eight types of phages but was also greatly attenuated in mice (Filippov et al., 2011). Our previous study revealed that the *waaA* null mutant lost most of its phage-binding activity in an adsorption assay while remaining sensitive to high titers of Yep-phi (Zhao et al., 2013). Crystal structure analysis of WaaA from *A. aeolicus* suggested that the N-terminal domain functions as the acceptor–substrate binding site for the lipid A precursor (Schmidt et al., 2012). In contrast, the waaA* mutant harbors a 9-bp in-frame deletion (_249_GTCATCGTG_257_, resulting in the _84_TMT_86_ deletion in WaaA) that is located at the junction of the N-terminal β2 sheet and α2 helix (**Supplementary Figure S2**). Basic amino acids on the N-terminal α2 helix have been shown to be crucial for facilitating the entry of the lipid A precursor into the acceptor–substrate binding site of WaaA (Schmidt et al., 2012). Here, we found that the phage-induced 9-bp in-frame deletion in *waaA* had similar effects to the *waaA* null mutation in *Y. pestis* regarding truncated LPS and phage resistance.

Previous research has revealed that *Yersinia pseudotuberculosis* with the *cmk* null mutation has a growth defect and is highly attenuated in mice (Walker et al., 2012). Our data indicate that the phage-induced cmk* mutation (with a _15_CCGGTGATAA_24_ frameshift deletion leading to the failure of Cmk translation) displays a similar phage-resistant phenotype as Δ*cmk*. However, how defects in *cmk* confer phage resistance to *Y. pestis* is unclear. Because T7-like phages depend on the nucleoside monophosphate kinase of the host (Qimron et al., 2006), we propose that the loss of Cmk impacts nucleoside synthesis pathways and hinders the replication of phages in the host. In this study, other genes encoding nucleoside monophosphate kinases, such as *adk*, *tmk*, *gmk*, and *pyrH* (Qimron et al., 2006), were found to be unaffected in the phage-resistant S56 strain, likely due to the high fitness costs associated with mutations in these essential genes. Notably, cmk* showed growth deficiency in addition to phage resistance, similar to waaA*. This finding suggests that bacterial resistance to lytic phages comes at a cost to growth fitness.

Previous research has demonstrated that laboratory passages of *Y. pestis* may result in the disruption or premature truncation of Ail expression (Leiser et al., 2015). Ail is a critical virulence factor of *Y. pestis*, playing a central role in promoting immune resistance to human host defense (Zhang et al., 2020; Kent et al., 2021). We have reported that ail* with a frameshift mutation in *ail* (A_538_ deletion) and Δ*ail* have similar phenotypes, indicating that the A_538_ deletion affects the functional integrity of Ail. Both waaA* and ail* attenuated phage adsorption, and the phage resistance of waaA*/ail* was additive compared with that of ail* or waaA* alone. Thus, we speculate that the phage primarily relies on LPS as an adsorption receptor, whereas *ail* is a secondary receptor, thereby affecting the reduction in viral progeny through different loadings onto host bacteria (Zhao et al., 2013).

The ail* mutation, which was observed only in S56 (the final passage), plays a minor role in phage resistance. However, the mutation in *ail* restores the growth of *Y. pestis* that was disrupted by a single mutation in *waaA* or *cmk.* In the phospholipid biosynthesis pathway, CTP and dCTP are downstream products of Cmk and may affect outer membrane lipids (Langley and Kennedy, 1978). In addition, the mutation in *waaA* can result in the accumulation of tetraacylated precursor lipid IV A (Tzeng et al., 2002). In contrast, the deletion of *ail* has been reported to cause an abnormal flow of phospholipids into the outer leaflet of the outer membrane (Kolodziejek et al., 2021), which might counterbalance the detrimental effects of accumulating mutations in *waaA* and *cmk*. One plausible explanation is that the *ail* mutation rebalances the changes in lipid content of the outer membrane caused by *waaA* and *cmk* mutations, thereby modifying cell shape and accelerating growth. Our data suggest that mutations in waaA* and cmk* may be advantageous to *Y. pestis* in the presence of phages but come at the cost of growth defects when phages are absent. Notably, waaA^*^ and *cmk* ^*^ could recover their fitness through the *ail* mutation.

**Figure 7:**
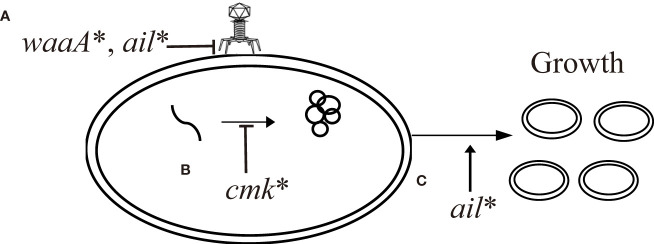
Proposed scenario of interplay between mutations in *Y. pestis* leading to resistance to Yep-phi phage infection. **(A)** Inhibition of phage adsorption to the surface of bacterial cells due to alterations in the surface receptor LPS by waaA* and ail*. **(B)** Inhibition of phage replication through secondary bacterial metabolites by cmk*. **(C)** Restoration of growth by ail*.

In this study, we identified successive mutations in *waaA*, *cmk*, and *ail* of *Y. pestis* that were induced by continuous phage challenges. Although these genes have individually been shown to be related to phage resistance in *Y. pestis* and other bacteria, they have not been observed simultaneously in one strain. Based on our findings, we propose a scenario in which *Y*. *pestis* developed a phage-resistant phenotype through mutations in *waaA*, *cmk*, and *ail* under continuous phage pressure. Initially, when phage stress was imposed on *Y. pestis*, the cells prevented phage adsorption through the *waaA* mutation, which truncates LPS. As phage pressure persisted, waaA*/cmk* mediated stronger phage resistance reinforced by the *cmk* mutation, which enhances resistance to phage lysis but leads to growth defects. Finally, the growth of waaA*/cmk* was restored by the *ail* mutation, and waaA*/cmk*/ail* prevented phage attachment because the Ail receptor was damaged. The interplay between mutations in the WaaA–Cmk–Ail cascade illustrates a tradeoff strategy during the development of phage-resistant phenotypes in *Y. pestis* (**Figure 7**).

All three genes responsible for phage resistance in *Y*. *pestis* discussed in this study are known to be virulence-related factors for pathogenic *Yersinia*. In the case of CO92, the *waaA* null mutant exhibited an LD50 nearly 4 million times lower than that of its wild-type counterpart (Filippov et al., 2011). In mice, the KIM5 *ail* null mutant showed > 3,000-fold increase in LD_50_ (Felek et al., 2010). The *cmk* null mutant in *Y. pseudotuberculosis* was > 400 times attenuated in the mice model (Walker et al., 2012). These findings suggest that phage pressure may have an impact on virulence and fitness in other niches of *Y. pestis*, which could partially explain the scarcity of natural phage-resistant isolates of *Y. pestis*.

A caveat to consider is whether these mutations are involved in antibiotic resistance in *Y*. *pestis*. Studies have suggested that LPS regulated by WaaA plays a role in polymyxin resistance, and an Ail-like protein imparts ceftriaxone resistance to *Salmonella enterica* (Hu et al., 2005; Moffatt et al., 2019). In *Y. pestis*, the minimal inhibitory concentration of polymyxin B for the *waaA* null mutant is 250 times lower than that of its wild-type counterpart, possibly due to the less efficient incorporation of 4-amino-4-deoxyarabinose into lipid A (Dentovskaya et al., 2011). Furthermore, reduced Cmk activity may increase the tolerance of *Staphylococcus aureus* to vancomycin through effects on cell wall biosynthesis (Matsuo et al., 2013). Notably, all these antibiotics target the bacterial membrane, which suggests that phage resistance likely relies on changes in membrane characteristics. Fortunately, there is no evidence linking the WaaA–Cmk–Ail cascade to resistance against first-line antibiotics used to treat plague (Barbieri et al., 2020). Studies using animal models and clinical studies have identified tradeoffs between resistance and virulence. These findings bode well for improving treatment effectiveness despite the development of phage resistance (Oechslin, 2018; Mangalea and Duerkop, 2020).

In this study, we examined the microevolutionary processes of *Y. pestis* under phage stress and found that phage exposure resulted in complex changes in *Y. pestis*, particularly perturbations of the cell membrane. The findings of the present study provide valuable insights into the molecular mechanisms underlying *Y. pestis* resistance to bacteriophage and may aid ongoing investigations into phage therapies for plague. Notably, as a facultative intracellular pathogen with a complex life cycle, *Y. pestis* is likely to develop phage resistance through different pathways under different conditions, such as in natural environments, fleas, and hosts if exposed to phage pressure. We acknowledge that these three genes (*waaA*, *cmk*, and *ail*) were identified through continuous *in vitro* selection with phage pressure and caution against using our data to predict or interpret what may happen in human or rodent hosts. Investigating the relationship between phage resistance and virulence characteristics of *Y. pestis* during long-term phage therapies will be valuable, although ethical and technical obstacles need to be addressed. Additional genetic factors related to phage resistance in *Y. pestis* should be discovered through parallel passages under phage pressure. Moreover, conditions more closely resembling the host environment may help identify pathways of phage resistance in *Y. pestis*.

## Data availability statement

The datasets presented in this study can be found in online repositories. The names of the repository/repositories and accession number(s) can be found in the article/**Supplementary Material**.

## Author contributions

LX, ZQ, and KS conducted the experiments, analyzed the data, and authored the paper. The phage adsorption experiments were carried out by KS and RL. HZ, HW, CL, and YX took part in the screening of phage-resistant strains. YJ, XL, XX, and XuZ contributed to the development curve of the 614F strain. RC, YT, ZD, and YC contributed to the interpretation of the data. RY, XiZ, and YS designed the experiments and contributed to the production and revision of the text. All authors contributed to the article and approved the submitted version.
