# Supplementary material for: Interplays of mutations in waaA, cmk, and ail contribute to phage resistance in Yersinia pestis
Source: Front Cell Infect Microbiol. 2023 May 26;13:1174510. doi: 10.3389/fcimb.2023.1174510 (PMC10254400; doi:10.3389/fcimb.2023.1174510)
Supplement: Supplementary file 1 [file DataSheet_1.docx]

Supplementary Material

Interplays of mutations in *waaA*, *cmk*, and *ail* contribute to phage resistance in *Yersinia pestis*

**Lisheng Xiao^1,2,3^**^†^**, Zhizhen Qi^4,5^**^†^**, Kai Song^3^**^†^**, Ruichen Lv^6^, Rong Chen^7^, Haihong Zhao^4,5^, Hailian Wu^4,5^, Cunxiang Li^4,5^, Youquan Xin^4,5^, Yong Jin^4,5^, Xiang Li^4,5^, Xiaoqing Xu^4,5^, Yafang Tan^3^, Zongmin Du^3^, Yujun Cui^3^, Xuefei Zhang^4,5^, Ruifu Yang^3,5^,** **Xilin Zhao^1^*, Yajun Song^2,3,5^***

*** Correspondence:** Yajun Song: songyj@bmi.ac.cn. Xilin Zhao: zhaox5@njms.rutgers.edu

# Supplementary Figures and Tables

## Supplementary Figures


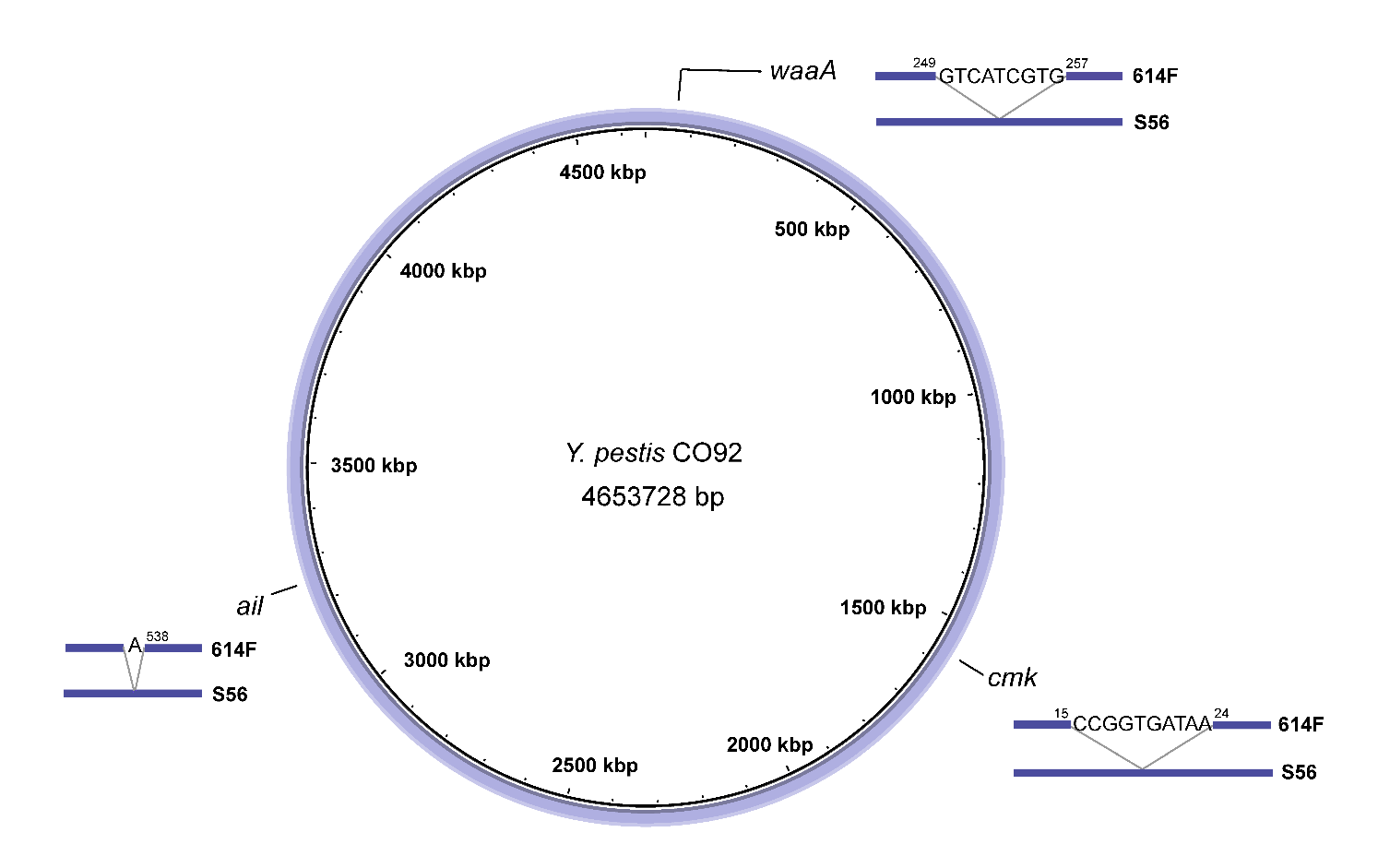


**Supplementary Figure 1.** **Mutations identified in S56 mutant strain**. Purple circle, the chromosome map of *Y. pestis* CO92 (accession: NC_003143.1). The markings on the circle indicate the genes (*waaA*, *cmk*, and *ail*) and locations mutated in S56, which are annotated according to CO92. “GTCATCGTG,” “CCGGTGATAA,” and “A” represent deletions of bases in mutant strain compared with 614F wild strain. The numbers on the markings indicate locations of mutant bases from the start of the gene.


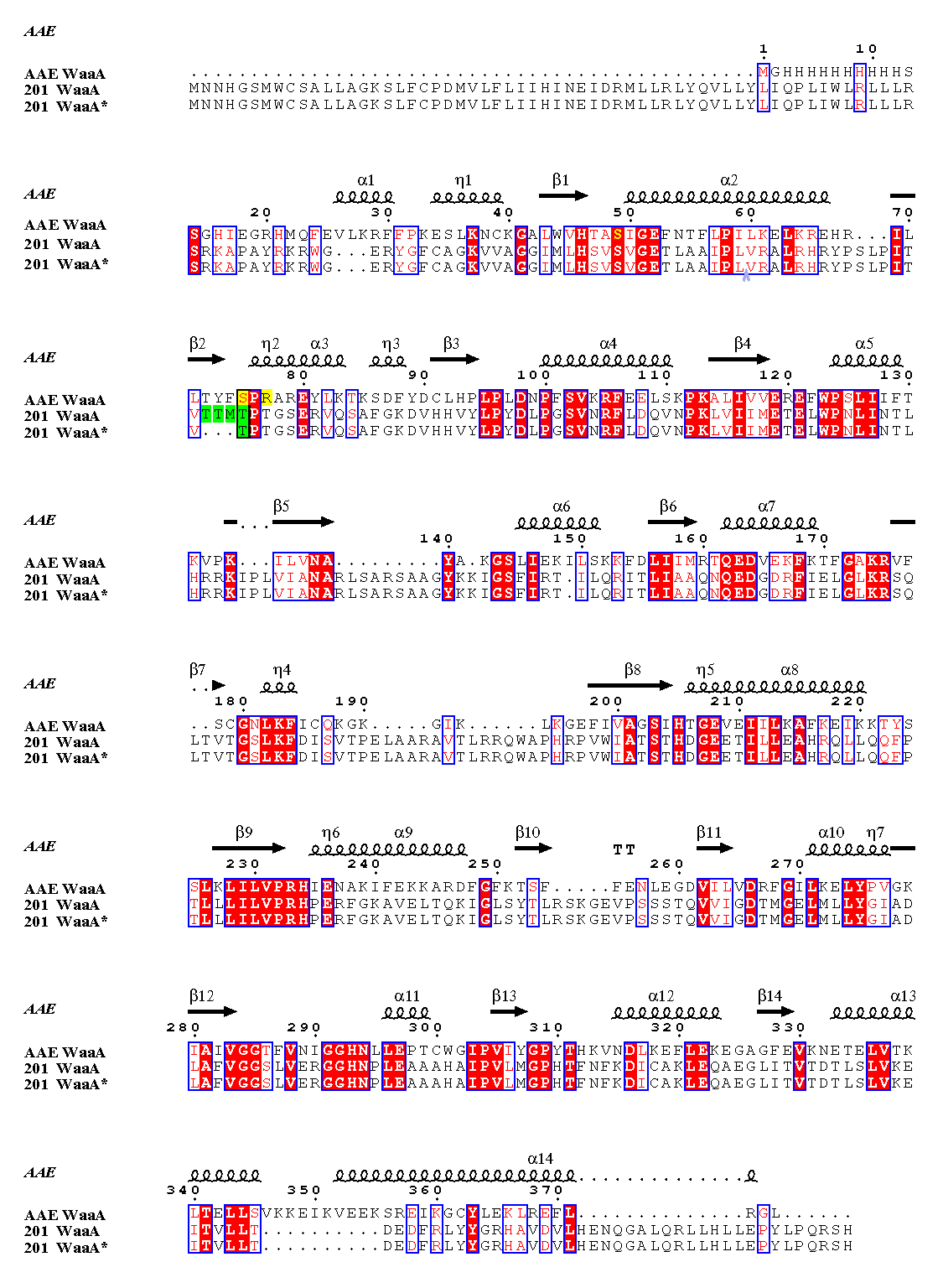


**Supplementary Figure 2.** **Sequence alignments and secondary structure prediction of *waaA* with *Aquifex aeolicus* template (PDB ID: 2XCI.A) using ESPript.** The _84_TMT_86_ deletion is highlighted in green. Functionally important sites (S54 and R56) of *Aquifex aeolicus* are marked in yellow.

**
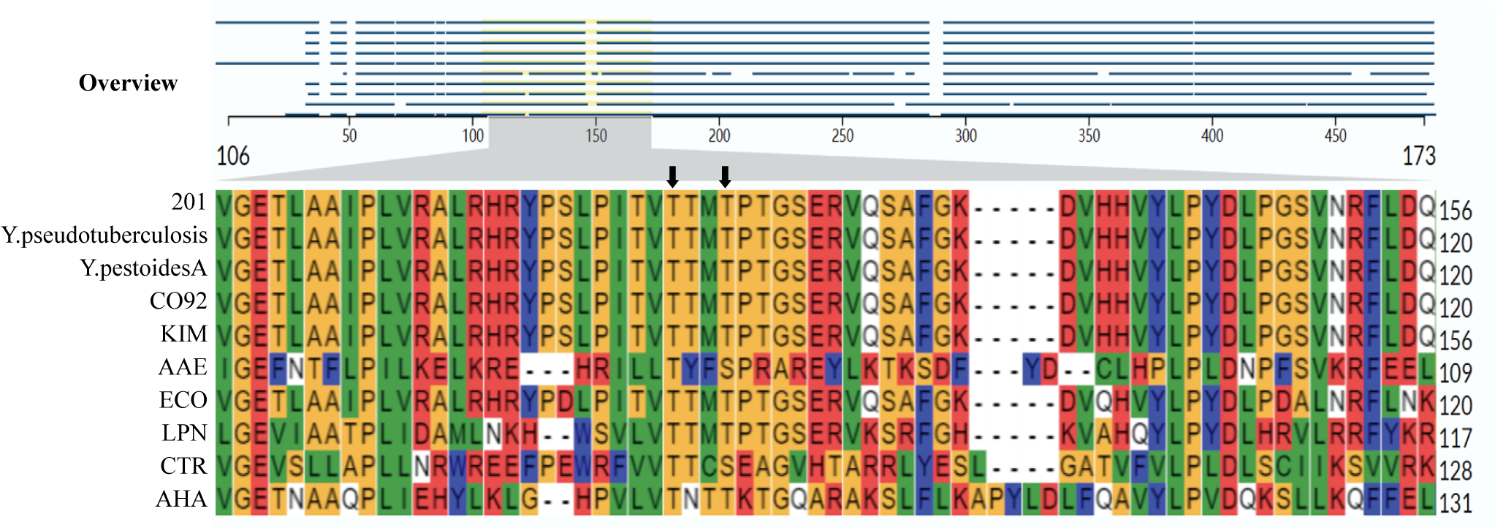
**

**Supplementary Figure 3.** **Comparison of the deduced amino acid sequence of *waaA* using UniProt for alignment.** CO92, *Y. pestis* strain CO92; KIM, *Y. pestis* strain KIM; AAE, *Aquifex aeolicus*; AHA, *Acinetobacter haemolyticus*; CTR, *Chlamydia trachomatis*; ECO, *Escherichia coli*; LPN, *Legionella pneumophila.*


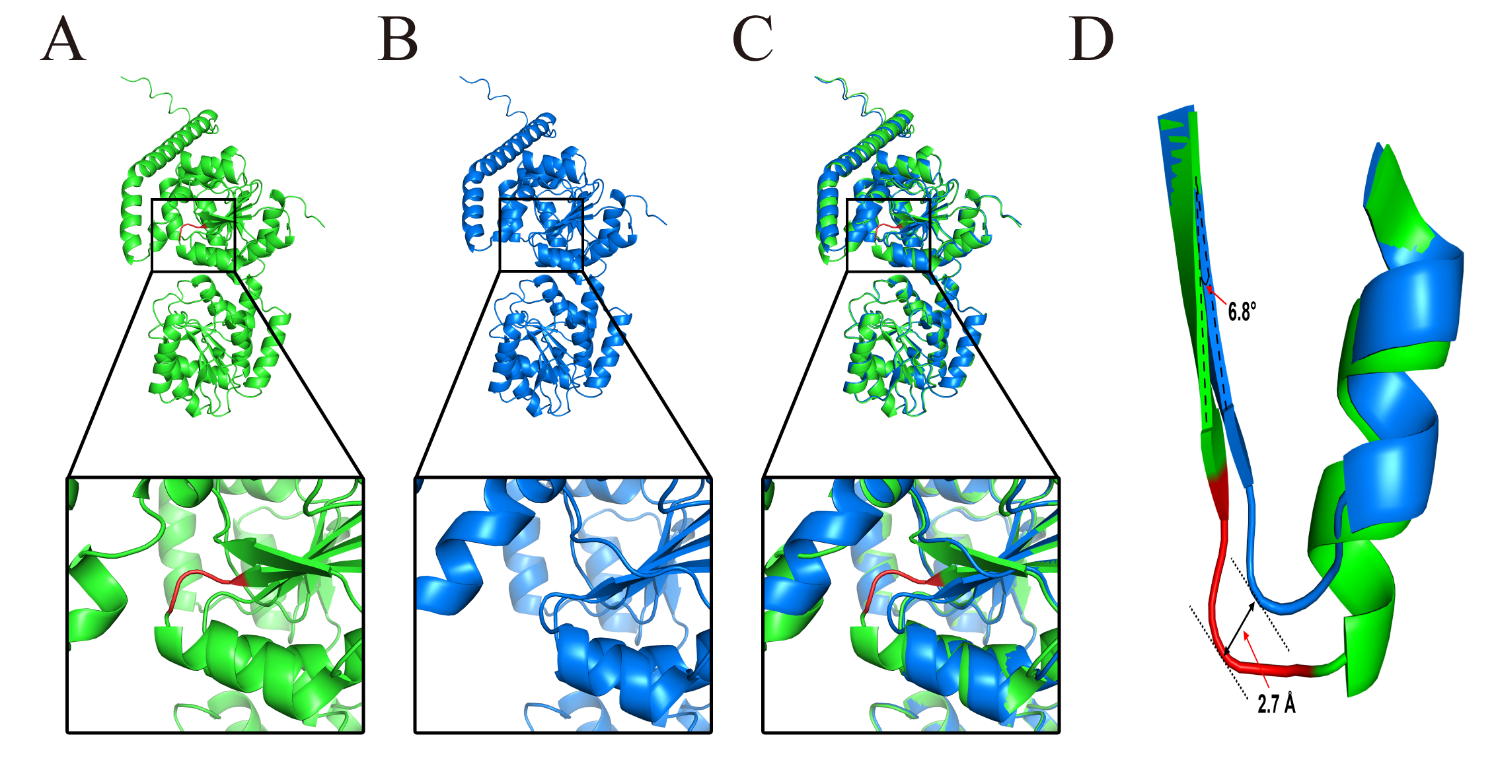


**Supplementary Figure 4.** **Predicted 3D model of *Y. pestis* WaaA and WaaA*** **proteins** **by AlphaFold.** **(A)** WaaA protein of *Y. pestis*. The _84_TMT_86_ fragment is shown in red. **(B)** WaaA* protein of *Y. pestis*. **(C)** Alignment of the predicted 3D structure of WaaA and WaaA* proteins of *Y. pestis* using PyMOL, RMSD = 0.37. **(D)** Comparing the α helix-loop-β turn structures of _84_TMT_86_ (in red) in WaaA protein (in green) and WaaA* (in blue) of *Y. pestis*, showing the distance and angle changes.

**
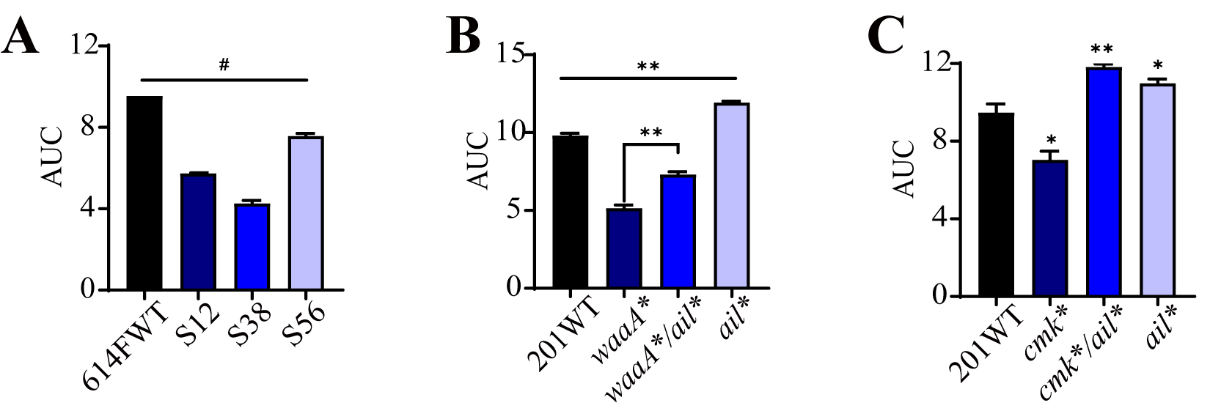
**

**Supplementary Figure 5.** ***ail** restored the growth of *waaA** and *cmk**.** Growth curves of *Y. pestis*WT and mutants cultured in LB for 12 h were analyzed using the area under curve (AUC). (*n* = 3, **P* < 0.05, ***P* < 0.01, ^#^*P* < 0.0001 compared to all other groups, Tukey’s multiple comparison test following ANOVA, mean displayed ± SEM).

## Supplementary Tables

**Table S1** ***E. coli* strains and plasmids used in this work**

| **Strains/plasmids** | **Characteristics** | **Source/reference** |
| --- | --- | --- |
| ***E. coli*** |  |  |
| S17-1 λpir | This strain has chromosomally integrated conjugal transfer functions | Lab collection |
| S17/pDS132_*waaA** | S17 harboring the *waaA* of S56 on pDS132; | This work |
| **Plasmids** |  |  |
| pMDISI | Spe resistance gene flanked by FRT and I-SceI sites; Spc^r^ Amp^r^ | (Kim et al., 2014) |
| pREDTKI | *ori* SC101(Ts) Kanr; *ara*BAD promoter for λ-Red; *trc* promoter for I-SceI; Kan^r^ | (Kim et al., 2014) |
| pKSI-1 | pBluescript II KS (-) backbone with I-SceI site–kanMX–I-SceI site cassette; Amp^r^, Kan^r^ | (Kim et al., 2014) |
| pKSI-1_*waaA** | Cloning vector with a PCR fragment covering a region from 337-bp fragment upstream to 295-bp fragment downstream of the *waaA* of S56 in the SacI/HindIII site; Amp^r^ | This work |
| pKSI-1_*cmk** | Cloning vector with a PCR fragment covering a region from 909-bp fragment upstream to 921-bp fragment downstream of the *cmk* of S56 in the SacI/HindIII site; Amp^r^ | This work |
| pKSI-1_*ail** | Cloning vector with a PCR fragment covering a region from 109-bp fragment upstream to 797-bp fragment downstream of the *ail* gene of S56 in the SacI/HindIII site; Amp^r^ | This work |
| pDS132_*waaA** | Cloning vector with a PCR fragment covering a region from 343-bp fragment upstream to 462-bp fragment downstream of the *waaA* of S56 in the SacI/SalI site; Cm^r^ | This work |
| pBAD33_*cmk* | Cloning vector with a PCR fragment covering a region from the *cmk* of 201 in the SalI/HindIII site; Cm^r^ | This work |
| pACYC184_*waaA* | Cloning vector with a PCR fragment covering a region from 500-bp fragment upstream to 300-bp fragment downstream of the *waaA* of 201 in the BamHI/HindIII site; Cm^r^ | Lab collection |
| pET32a_*waaA* | Cloning vector with a PCR fragment covering the *waaA* of 201 in the BamHI/HindIII site; Amp^r^ | This work |
| pET32a_*waaA** | Cloning vector with a PCR fragment covering the *waaA* of S56 in the BamHI/HindⅢ site; Amp^r^ | This work |

**Table S2** **Primers used in this work**

| Target | Primer (forward/reverse,5'-3') |
| --- | --- |
| Mutant construction | |
| Δ*waaA* | TATGGTTTTTGTGCCGGTAAAGTCGTTGCTGGCGGTATCATGCTGCATTCCGTTTCAGTCCCTTCCCGGCGATCCTCTGG/GATCAGGTTAGGCCAAAGTTCAGTTTCCATGATGATAACCAGTTTGGGATTGACCTGGTCCCCGCATGACGGCAAGTGGACG |
| pKSI-1_*waaA** | TCCGAGCTCTGTGGTGCTCCGCCCTCCTA/CCCAAGCTTGTTCTGTGCCGCAATCAA |
| Δ*cmk* | CTAACTGCTGCAGATATTGGTACCTACGATGACCACCGTATGGCGATGTGTTTCTCGCTGCCCTTCCCGGCGATCCTCTGG/TTCGTTGGGCATAAGCCAGCGCCTGTTCGATCACCTGTTCGATGGACATACTGGTTGAATCCCGCATGACGGCAAGTGGACG |
| pKSI-1_*cmk** | TCCGAGCTCGGGACTTATTTGGTTGAGG/CCCAAGCTTACACCACGGACGATAGAG |
| Δ*ail* | TTACAATTTAATCCACATCCAAATTTTGTCATTGATGCTTCATATGAATACTCCAAACTCCCCTTCCCGGCGATCCTCTGG/TGACAAACTTCACGTTAAAAAATCGTCTATGAGCCAGAAGCAGCCCGGTATTCATTGGTGCCCGCATGACGGCAAGTGGACG |
| pKSI-1_*ail** | TCCGAGCTCCGGTGCCGGATTACAATT/CCCAAGCTTCGCAGAGCCGATAACAAA |
| pDS132_*waaA** | CGAGCTCATGAATAACCACGGCTCA/GCGTCGACGTGCTAGTCGCAATCCAA |
| pBAD33_*cmk* | GCGTCGACATGACGGCGATAGCCCCG/CCCAAGCTTTTATTTTTTCAACGGCAA |
| Verification of the mutants | |
| Δ*waaA* | GAATAACCACGGCTCAAT/CAACGGGAATCCTGCTCT |
| pKSI-1_*waaA** | CACCCAACAGCTCTAACG/GGCCACGGATGCCAAACA |
| *waaA** | AGCACCCAACAGCTCTAA/ATACCGCCAGCAACGACT |
| Δ*cmk* | TACCGCTCACCAGGGACT/CCGTAACCAGCAAATCAA |
| pKSI-1_*cmk** | GGGTCGAGGTGCCGTAAA/TGCCGTAGTCGCAATGGT |
| *cmk** | GGGGCATGAGAACTACCA/GCTCTTTGCCTTCCAGAT |
| Δ*ail* | TTCCGCATTAACGAGTATGT/CCGTAACCAGCAAATCAA |
| pKSI-1_*ail** | CGTCCCATTCGCCATTCA/AAGCCCAGCCGTTACCAA |
| *ail** | CCTTCAACCGTATCAACTC/CTCGGATAATAGAACCTTCC |
| pDS132_*waaA** | TGTGAGCGGATAACAATT/CAGTGCGGTAGTAAAGGT |
| pBAD33_*cmk* | ATGCCATAGCATTTTTATCC/GATTTAATCTGTATCAGG |
| verification of overexpress-*cmk* | |
| Target (*cmk*) | CGAGTGGTGCGGGTAAAG/AGATGTGCGGCAAGTGGT |
| Reference (16S) | GCCACACTGGAACTGAGACACG/CGCTGAAAGTGCTTTACAACCC |
| Protein expression | |
| *waaA* | CGCGGATCCATGAATAACCACGGCTCA/CCCAAGCTTTTAGTGGCTCCGTTGTGG |
| *waaA** | CGCGGATCCATGAATAACCACGGCTCA/CCCAAGCTTTTAGTGGCTCCGTTGTGG |
| Complementation of mutants | |
| *waaA** | CGCGGATCCGTGCAAAAGCCGTAGTC/CCCAAGCTTCGATTTTAAGCTCTGGG |
| Verification of gene deletion in 614F | |
| *waaA** | CTGCCTATCACGGTGACG/CGATAACGACTTGGGTGC |
| *cmk** | AATGGCGAGGAGTAAGCG/CTCGGTCCATCAACGGAG |
| *ail** | GGATGCTTGGCGCGGGCA/TAGCATTGGAATAAGCTCG |

Underlining indicates endonuclease sites.
